# A Multifaceted Approach to Optimizing AAV Delivery to the Brain for the Treatment of Neurodegenerative Diseases

**DOI:** 10.3389/fnins.2021.747726

**Published:** 2021-09-24

**Authors:** Jonathan M. Fischell, Paul S. Fishman

**Affiliations:** Department of Neurology, University of Maryland School of Medicine, Baltimore, MD, United States

**Keywords:** gene therapy, genetic vectors, neurodegenerative disease, intra-arterial (IA) delivery, intra-thecal drug delivery systems, adeno-associated virus (AAV), blood–brain barrier disruption, capsid engineering

## Abstract

Despite major advancements in gene therapy technologies, there are no approved gene therapies for diseases which predominantly effect the brain. Adeno-associated virus (AAV) vectors have emerged as the most effective delivery vector for gene therapy owing to their simplicity, wide spread transduction and low immunogenicity. Unfortunately, the blood–brain barrier (BBB) makes IV delivery of AAVs, to the brain highly inefficient. At IV doses capable of widespread expression in the brain, there is a significant risk of severe immune-mediated toxicity. Direct intracerebral injection of vectors is being attempted. However, this method is invasive, and only provides localized delivery for diseases known to afflict the brain globally. More advanced methods for AAV delivery will likely be required for safe and effective gene therapy to the brain. Each step in AAV delivery, including delivery route, BBB transduction, cellular tropism and transgene expression provide opportunities for innovative solutions to optimize delivery efficiency. Intra-arterial delivery with mannitol, focused ultrasound, optimized AAV capsid evolution with machine learning algorithms, synthetic promotors are all examples of advanced strategies which have been developed in pre-clinical models, yet none are being investigated in clinical trials. This manuscript seeks to review these technological advancements, and others, to improve AAV delivery to the brain, and to propose novel strategies to build upon this research. Ultimately, it is hoped that the optimization of AAV delivery will allow for the human translation of many gene therapies for neurodegenerative and other neurologic diseases.

## Introduction

Gene therapies provide several major advantages compared to all other therapeutics which make them uniquely valuable for the treatment of neurodegenerative diseases (NDDs). First, gene therapies must be delivered intracellularly in order to function (Ingusci et al., 2019). Though this poses a challenge, once successfully delivered, gene therapies are able to directly modify protein production at the source (Ingusci et al., 2019). This is especially advantageous for the treatment of NDDs as the vast majority of pathology in these conditions occur intracellularly (Kovacs, 2019). Second, the vast majority of NDDs are caused by the dysfunction of a single gene, or small number of pathogenic proteins (Kovacs, 2016, 2019), either of which can be directly targeted by gene therapies. Finally, DNA-based gene therapies are adopted by host cells and can reside in the nucleus where they act as a blueprint, allowing cells to produce a therapeutic protein(s) continuously without re-administration (Nathwani et al., 2014). For this reason, DNA-based gene therapies should only require a single dose to give long lasting, or even life-long disease modification. This is particularly valuable for neurologic diseases as the blood–brain barrier (BBB) complicates delivery of large therapeutics to the brain, often requiring more invasive routes of delivery.

Unfortunately, the delivery of gene therapies to the brain faces significant challenges. Delivering genes to the cells in the brain is difficult, as DNA is a large, charged, molecule, and will not penetrate the BBB and cellular membrane (Maeder and Gersbach, 2016). In order to facilitate delivery of gene therapies to the intracellular compartment of target cells, a vector must be employed. Vectors may be non-viral or viral. Non-viral vectors include lipid nanoparticles (Bors and Erdö, 2019), polymer nanoparticles (Liu et al., 2017), cell penetrating peptides (Liu et al., 2017), and cationic microbubbles [used with focused ultrasound (FUS)] (Fan et al., 2016). The major benefit of non-viral vectors is that they lack capsids, which are made up of foreign proteins and therefore are immunogenic, albeit to varying degrees (Bessis et al., 2004). Though non-viral vectors do not possess immunogenic capsids, the risk of immune response to the delivered *transgene* remains a major problem (Mulia et al., 2020; Ediriweera et al., 2021). Additionally, transgenes, themselves may be toxic to cells through insertion into the host’s chromosomal DNA (insertional mutagenesis) (Cavazzana-Calvo and Fischer, 2007) or undesired alteration of off target protein expression (Balakrishnan et al., 2013). Additionally, even intended manipulation of protein expression could theoretically result in deleterious effects. For example, knocking down the pathogenic protein in a NDD may improve disease phenotype (Alarcón-Arís et al., 2020), however, in many cases it remains unclear if that protein also serves a vital cellular function in humans (Brundin et al., 2017; Wild and Tabrizi, 2018). If that were the case, unintended side effects of gene therapy would be likely. The greater the number of cells transduced, the greater the likelihood of immune or non-immune mediated side effects thus minimization of off target delivery is critical to the safety of both non-viral and viral vector mediated gene therapy. In order to minimize off target delivery, vectors should be targeted to cells involved in disease pathology. Altering both the tissue and cellular tropism of gene therapy vectors will likely be necessary to make the safest gene therapy product possible. Owing to their simplicity of design, precise alteration of tropism is not yet possible with non-viral vectors (Liu et al., 2017).

Viral capsids have evolved for over a billion years to be able to effectively allow the deposition of their genetic contents intracellularly, and even to the nucleus of cells. By comparison, non-viral solutions are more simplistic, and thus are less effective at delivering their cargo intracellularly (Liu et al., 2017). Furthermore, viral vector capsids are made of proteins, which, made from DNA blueprints, are modifiable to suit varied purposes (Hudry and Vandenberghe, 2019). Over the past 30 years, many viral vectors have been developed for gene therapy to the central nervous system (CNS), including lentivirus, adenovirus and adeno-associated viruses (Agustín-Pavón and Isalan, 2014; Bedbrook et al., 2018; Hudry and Vandenberghe, 2019; Ingusci et al., 2019). Due to the low immunogenicity, high efficiency, high stability and ease of modification, AAV vectors have become the mainstay delivery tool for gene delivery to the CNS (Choudhury et al., 2016; Lykken et al., 2018). A large variety of serotypes, each with different cellular and tissue tropism have been discovered thus far (Zincarelli et al., 2008; Snyder et al., 2011; Hocquemiller et al., 2016; Hudry and Vandenberghe, 2019; Keeler and Flotte, 2019). Owing to their simple genetic composition (of only three viral proteins named VP1, VP2, and VP3), modifications to the capsid of AAVs is easily implemented (Hocquemiller et al., 2016; Deverman et al., 2018). The only major downside of AAVs, is that their small size limits the size of cargo which can be delivered by a single vector (Bedbrook et al., 2018). Though this poses a problem, there is extensive work being done to develop compact transgenes capable of fitting in an AAV without altering function. For example, one group designed a CRISPR-dCas9 delivery system capable of fitting in a single AAV vector by using a smaller Cas9 variant from *Staphylococcus aureus rather* than the typical *Streptococcus pyogenes* (Lau et al., 2019). Ultimately, AAVs provide a valuable tool for gene therapy. It is not surprising that the first approved single dose gene therapy, Onasemnogene abeparvovec-xioi (AVXS-101; or Zolgensma^®^) for spinal muscular atrophy (SMA), uses an AAV (specifically AAV9) (Mendell et al., 2017).

Armed with vectors such as AAVs, and with platforms capable of making highly specific alterations to gene expression (Larson et al., 2013; Agustín-Pavón and Isalan, 2014; McMahon and Cleveland, 2016; Savić and Schwank, 2016; Lau and Suh, 2017; Lau et al., 2019; Lim et al., 2020), we are closer to a reality where gene therapy platforms capable of correcting the underlying cause of many NDDs. These diseases include disorders caused by dysfunction of a single gene [e.g., Huntington’s (HD) (Wild and Tabrizi, 2018), Spinocerebellar ataxias (SCAs) (Dong and Cong, 2019; Martier et al., 2019), and lysosomal storage disorders (LSDs) (Ohashi, 2019)]. This approach may also be applicable to treat more common NDDs which are considered multifactorial or polygenic, but result from the toxic accumulation of a single, or small number of, proteins [e.g., Alzheimer’s disease (AD) (Elmer et al., 2019), Parkinson’s disease (PD) (Brundin et al., 2017), and Amyotrophic Lateral Sclerosis (ALS) (Scotter et al., 2015)]. Even if these platforms become available, specific and efficient delivery strategies will likely be necessary for clinical translation.

For a gene therapy to be effective, it must transduce enough target cells to mitigate the disease pathology. Theoretically, one way to achieve this would be to increase the dose of virus until a sufficient level is reached. However, the ability to do this is prevented by dose dependent toxicities which are largely mediated by the immune system (Rabinowitz et al., 2019; Perez et al., 2020; Verdera et al., 2020). The broad translation of gene therapy is contingent upon maximizing gene delivery to target cells, while minimizing the risk of adverse events associated with off-target delivery. A historical example of when this went awry is the death of Jesse Gelsinger in 1999. In this case, Jesse was treated for ornithine transcarbamylase deficiency with an IV adenoviral vector for gene replacement. Tragically, he died 4 days later of a massive immune response and multisystem organ failure (Sibbald, 2001). Though gene therapy has come a long way since this time, similar responses have been seen in non-human primates (NHPs) and AAVs, delivered IV, result in hepatotoxicity (Bessis et al., 2004; Mingozzi and High, 2017; Fitzpatrick et al., 2018), while direct CNS delivery can result in neurotoxicity (Samaranch et al., 2014; Meyer et al., 2015; Hordeaux et al., 2018a; Perez et al., 2020).

AVXS-101 is the first approved single dose gene therapy for a neurologic disease (SMA type 1) (Mendell et al., 2017). Though the benefits of this treatment are clear, it is worth noting that significant elevations in the liver enzymes (AST and ALT) have been seen in a large fraction of treated patients (Mendell et al., 2017), signifying subclinical hepatotoxicity. Though the children in the AVXS-101 trial did not develop serious injury from this treatment, the incidence of transaminitis and the necessity co-administration of steroids, suggests that this trial used a near maximum tolerable dose in humans. Further, the sample size in this study was small, Thus, with more subjects, more serious reactions may have been observed. Finally, infants with SMA type 1 are ideal candidates for gene therapies as they have a less mature immune system (Simon et al., 2015), healthy organs, and are unlikely to have developed neutralizing antibodies to AAVs (Fitzpatrick et al., 2018). When it comes to the broad translation of gene therapy for more common neurologic diseases which effect adults (e.g., AD or PD), products with a higher margin of safety are necessary.

There are many gene therapies for NDDs which have shown promise in animal models and are ready for translation to humans (Yang et al., 2017; Arrant et al., 2018; Martier et al., 2019; Sun and Roy, 2021). However, many of these therapies use naturally occurring AAV vectors delivered via inefficient delivery routes and are expressed with non-specific promotors. The utilization of more advanced delivery technologies, which optimize on-target delivery, will likely be necessary to develop safe, effective and broadly translatable therapies.

**FIGURE 1:**
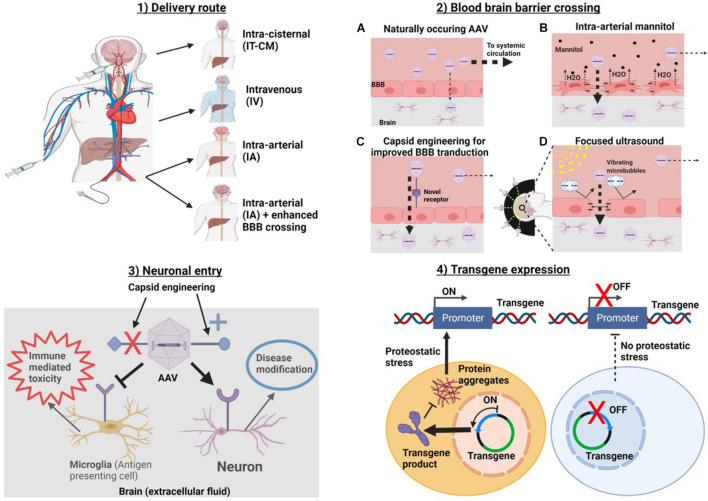
Methods for optimizing at each of four stages of AAV delivery to the brain. In order to get to the brain AAVa are **(1)** introduced into the body via a delivery route. Routes which avoid systemic delivery such as IT-CM and IA with BBB crossing should be optimal. **(2)** If intravascularly delivered, a strategy for enhancing BBB delivery should be chosen. **(3)** Target cell entry (i.e., neurons for NDDs). This can be accomplished by capsid engineering to enhance specific cellular tropism. And **(4)** transgene expression. Promoters can be used to target transgene expression to specific cells and/or conditions, in this example, a promoter which drives gene expression in the context of proteostatic stress is used. This should reduce transgene toxicity. Created with Biorender.Com.

The process of delivery of AAVs to the brain can be divided into four major stages: (1) the delivery route, (2) BBB crossing, (3) neuronal entry, and (4) transgene expression (Figure 1). Thorough optimization of AAV delivery to the brain should consider each of these stages individually. Methods such intra-arterial delivery (Liguore et al., 2019), physical methods of transient BBB disruption (BBBD) (Foley et al., 2014; Fishman and Frenkel, 2017), AAV capsid engineering (Deverman et al., 2016) and cell-specific promotors (Jackson et al., 2016) have all been shown significantly improve the efficiency of gene therapy delivery, yet none of these methods are being investigated in human studies^1^. In our view, the ideal gene therapy for most NDDs of the brain has the following characteristics: (1) global delivery (2) single dose (3) minimally invasive (does not requiring opening skull) and (4) cell specific delivery and gene expression. This manuscript seeks to review a series of recent technological advancements shown to be effective in pre-clinical models, which could facilitate the development of this ideal therapy. It is our hope to provide a guide to methods that could be implemented, in humans, which would allow a translation of gene therapies for the treatment of many NDDs.

## Traditional Routes of Gene Therapy Delivery

Receiving a single dose gene therapy is a life altering event for a patient that poses both significant risk and significant potential benefit. Delivery route has a profound effect on vector biodistribution after injection. Altering this variable can enhance efficiency (off-target divided by on-target delivery) by a factor of tens, hundreds, or even thousands fold (Hinderer et al., 2018a; Marshall et al., 2018; Liguore et al., 2019; Bailey et al., 2020). A major limiting factor preventing the translation of gene therapies to the clinic is dose dependent toxicity which is directly related to high off-target delivery. For this reason, it is critical to use all tools at our disposal to optimize the delivery route for single dose gene therapies to the brain.

The determination of an optimal delivery route is dependent on the tissues involved in disease pathology. Many diseases affect the entire body, as well as the brain [e.g., LSDs (Filocamo and Morrone, 2011)]. As delivery outside the brain may improve disease pathology in these systemic diseases, a greater tolerance for routes with high systemic delivery, such as intravenous (IV), could be considered. Conversely, most NDDs, including as PD (Kovacs, 2019), AD (Kovacs, 2019), HD (Dong and Cong, 2019), and frontotemporal dementia (FTLD) (Svetoni et al., 2016) involve the entire brain, but are not known to pose a significant threat to the rest of the body. In these cases, an optimal route minimizes systemic delivery, while allowing for global delivery throughout the brain. These goals are often at odds with one another, as routes which are capable of delivering vectors evenly throughout the brain are also more likely to deliver vectors to the rest of the body. Finally, for all routes, the invasiveness of delivery must be considered.

### Evaluation of Direct Intracerebral Delivery

Historically, single dose gene therapies have been delivered to the brain by direct direct intracerebral injection (dIC) injection (Bilang-Bleuel et al., 1997; Mandel et al., 1997; Sanftner et al., 2004; Yang et al., 2017; Ingusci et al., 2019). This route was selected because large molecules, such as viral vectors and genes, could not cross the BBB at high enough concentrations to be effective (Rosenberg et al., 2018; Hudry and Vandenberghe, 2019). A major limitation to this route is its invasiveness. This route requires opening the skull and inserting a needle through the brain parenchyma to reach the injection site, a procedure known to carry a risk of intracerebral hemorrhage and CNS infection (Marks et al., 2010; Fugate, 2015). Many studies investigating the safety of dIC injection of AAVs report relatively low complication rates and found their treatments to be well tolerated (LeWitt et al., 2011; Bartus et al., 2013; Tardieu et al., 2014; Palfi et al., 2018). That said, is important to also recognize morbidity of open neurosurgical interventions which may not be quantified as a major adverse event, such as fear, pain and non-specific consequences of destroying brain tissue on the way to the target.

A second major limitation of dIC delivery is that it only delivers AAVs to a small area(s) of the brain. This poses a problem when treating NDDs, which effect the entire brain and/or spinal cord. Direct injection of AAVs to a specific brain region would only improve clinical manifestations caused by degeneration of that region. Additionally, it is now well established that pathogenic proteins engage in prion-like spread from brain cell to brain cell in many NDDs (Pearce, 2017). If disease can spread from one region to another, similar to an infection, disease burden must be viewed globally and therefore the effectiveness of treatment would be proportional to the volume of treated tissue.

So far, studies investigating dIC injection for the delivery of gene therapy in humans have been universally unsuccessful, citing inadequate distribution volume as a major limitation (Marks et al., 2010; Sudhakar and Richardson, 2019). To combat this problem, there is a significant amount work being done to develop methods for improving viral distribution after dIC injection (Piguet et al., 2021). Convection-enhanced delivery (CED) is one method being evaluated to enhance delivery (Sudhakar and Richardson, 2019). While this and other technologies may significantly improve the problem of distribution, they will not alter the invasiveness. Although a certain level of invasiveness could be tolerated for a single dose gene therapy, it would only be worth the risk if the invasive method was otherwise the better than any other method. As things currently stand, intrathecal (IT) delivery accomplishes the same goal as dIC delivery (injecting past the BBB), but does so less invasively and more globally.

### Evaluation of Intravenous Delivery and Meta-Analysis of Pre-clinical Studies

Intravenous delivery of gene therapy is appealing for because it is simple and non-invasive. After injection, IV gene therapies are not directed to the brain, and instead distribute throughout the body. In fact, owing to the BBB (Bors and Erdö, 2019), IV infusion favors delivery to all tissues besides the brain. This fact may be less of a limitation in systemic diseases with CNS manifestations, such as LSDs, but for most NDDs, where key manifestations are limited to the brain, this is suboptimal.

For an AAV to reach the brain after intravascular delivery, a strategy must be employed to facilitate its passage across BBB. This strategy could simply be that the AAV itself is capable of crossing. There are several naturally occurring AAVs known to cross the BBB, AAV9 (Samaranch et al., 2011; DiMattia et al., 2012; Tanguy et al., 2015; Saraiva et al., 2016), AAVrh8 (Yang et al., 2014; Hocquemiller et al., 2016), AAVrh10 (Yang et al., 2014; Tanguy et al., 2015; Rosenberg et al., 2018), and AAVhu.32 (Yoon et al., 2020). These vectors have surface receptors which allow them to enter endothelial cells and undergo transcytosis exiting on the other side of the BBB (Weber-Adrian et al., 2017). This mechanism allows for higher brain penetration than vectors, which do not possess this trait (Zincarelli et al., 2008). Although AAV9 and other naturally occurring vectors cross the BBB, they do so with low efficiency and many vectors have been engineered to improve BBB crossing properties (Chan et al., 2017; Hanlon et al., 2019). This will be discussed in more depth later in the manuscript.

**FIGURE 2:**
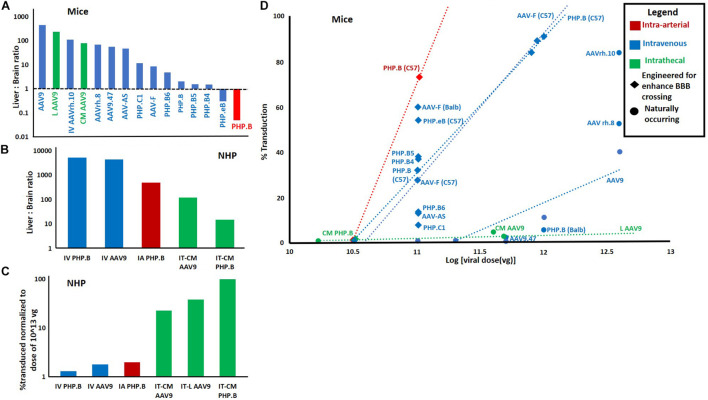
Meta-analysis of 14 papers reporting quantitative metrics of transduction efficiency using different routes and AAV serotypes. **(A)** Liver: brain ratio by route and serotype in mice. 26 data points from 12 studies in mice. Each used qPCR to report viral genome concentration in both liver and brain. Viral concentration in liver was divided by viral concentration in brain after injection of a single dose via IV, IT, or lA routes. **(B)** Liver: brain ratio in non-human primates. Eight data points from four studies in non-human primates. Calculated using same method as “A.” *For bath panels* **(A,B)** *the greater the ratio, the less efficient the route/serotype. combination*. **(C)** Percent transduction in NHPs normalized to a median dose of 10^13^. Percent transduction was calculated either by% GFP positive cells reported by authors or by estimating the percentage of brain cells transduced multiplying the number of viral genomes per cell calculated by authors via qPCR by 100 (i.e., if the ratio of viral genomes to normal cells is 1 then it was estimated that all cells or 100% of cells were transduced). This was then normalized to the median dose of l × l0^13^ vg by dividing the % transduction by the ratio of actual dose/median dose. Data was extrapolated from 10 data points from 4 studies in non-human primates. **(D)** % transduction (calculated by the same method as in panel **C**) vs. viral dose for different combinations of routes and serotypes. In the event that there the same route/serotype was used at different concentrations, the linear regression of these values is displayed as a dotted line with this line labeled, rather than each data point. This data was extrapolated from 31 data points from 11 studies was used. In panels **(A–D)** if there were multiple data points for a given route/serotype combination (and in panel **(D)** route/serotype/dose combination) the average of all data points was used.

Figure 2 is a meta-analysis of 12 published mouse studies (Pulicherla et al., 2011; Yang et al., 2014; Choudhury et al., 2016; Chan et al., 2017; Hordeaux et al., 2018a, b; Marshall et al., 2018; Hanlon et al., 2019; Huang et al., 2019; Lim et al., 2019; Bailey et al., 2020; Kumar et al., 2020) and 4 studies in NHPs (Yang et al., 2014; Hinderer et al., 2018a; Hordeaux et al., 2018a; Liguore et al., 2019). These studies used a variety of route and serotype combinations for gene delivery to the brain. Though these are not the only studies to have done this, this set studies were chosen because their histological results were reported in a way which allowed estimation of the percent of transduced cells.

This meta-analysis illustrates several key points regarding deliver of AAVs to the brain which we would like to highlight. The first is that IV AAV9 (the only approved combination of serotype and route for human gene therapy), appears to be very inefficient in both mice and NHPs. Quantitatively, in mice, only an average of 0.0008% (Yang et al., 2014; Choudhury et al., 2016; Marshall et al., 2018) of the total dose of AAV9 vectors transduced neurons. And, an average of 450× (Pulicherla et al., 2011; Yang et al., 2014; Choudhury et al., 2016; Hordeaux et al., 2018b; Marshall et al., 2018) more vectors were delivered to the liver, the primary site of known dose-dependent immune toxicity. This problem was significantly improved by engineered vectors with the ability to cross the BBB.

Secondly, one paper demonstrated that the intra-arterial (IA) route significantly enhanced brain, and reduced liver delivery of AAV PHP.B (Liguore et al., 2019). This was consistent another study with AAVhu.32, however, their data was not usable in meta-analysis (Yoon et al., 2020). The advantage of the IA route, over IV, was no longer present when investigating AAV PHP.B in NHPs (Figure 2). However, this may be explained by the fact that the vector used (PHP.B) does not efficiently cross the BBB in NHPs. The last finding from the meta-analysis to highlight is that the IT routes performed the best in NHPs but not in mice (Yang et al., 2014; Marshall et al., 2018; Bailey et al., 2020). This finding supports the potential of using this route in humans.

**FIGURE 3:**
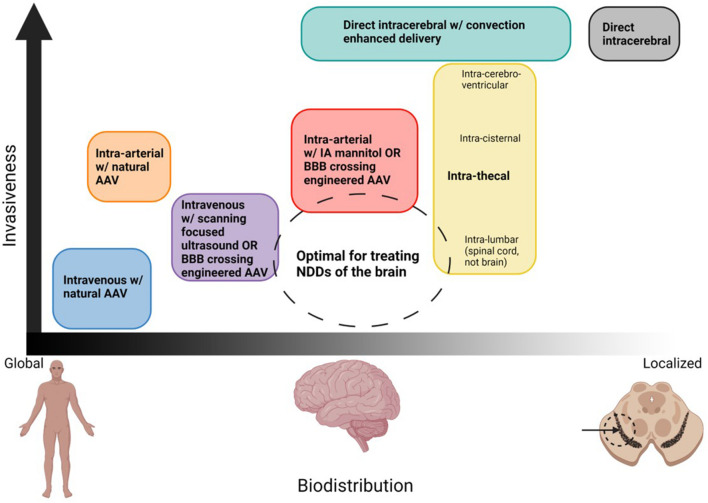
Organization of potential routes of AAV delivery based on biodistribution and invasiveness. Ideal routes for the treatment of NDDs would transduce the entire brain but minimize systemic delivery, and would the least invasive possible. The dashed circle denotes where this ideal treatment would lie. Although no delivery method is perfect, the closest would be intravenous or infra-arterial with a strategy to cross the BBB which works in humans, intra-cisternal or intra-lumbar for spinal cord diseases. Created with Biorender.Com.

In the distant future, AAVs may be developed which have far superior safety profiles. At that time, it may not be necessary to augment tissue specificity with delivery route. However, as things currently stand, AAV delivered gene therapies pose great potential risks, and as such, we should use all tools at our disposal to reduce these risks. Choice of delivery route provides a relatively easy way to enhance delivery efficiency and should not be ignored. Figure 3 estimates the compares the estimated biodistribution and invasiveness of various delivery route, indicating where an ideal route may lie.

## Intra-Thecal Delivery Routes

Intra-thecal (IT) delivery includes any delivery method where AAVs are injected into the subarachnoid space. This delivery can occur anywhere along the neuro-axis including intra-lumbar (IT-IL) via classic lumbar puncture between L4 and L5, intra-cisternal (IT-CM), via direct infusion into the basal cistern *via* sub-occipital puncture, and intracerebroventricular (IT-ICV), via direct injection through the skull into the lateral ventricle (Piguet et al., 2021). IT delivery is a promising method for the delivery because it naturally bypasses the BBB. In NHPs, IT delivery has been shown to be the most efficient route capable of global delivery to the brain (Yang et al., 2014; Hinderer et al., 2018a; Hordeaux et al., 2018a; Liguore et al., 2019). IT-CM delivery has also been shown to allow highly efficient delivery in mice, even demonstrating disease modification in a mouse model of GM1-Gangliosidosis (Hinderer et al., 2020; Chatterjee et al., 2021). As expected with IT therapy, there is considerably less peripheral uptake to all organs, including the liver (Yang et al., 2014; Hinderer et al., 2018a; Hordeaux et al., 2018a; Liguore et al., 2019). Thus, this route is expected to have considerably lower risk of hepatotoxicity compared to IV delivery. IT-CM delivery is under investigation in humans for several NDDs of the brain including FTLD (NCT04408625) and PD with at least 1 GBA mutation (NCT04127578).

Although IT delivery offers a promising solution to improve delivery throughout the brain, there are challenges with this approach. The first challenge is the concern for CNS toxicity with high doses. It has been demonstrated in NHPs and piglets, respectively that delivery of high doses of AAVs, IT, risks of dorsal root ganglion (DRG) toxicity (Hinderer et al., 2018b; Hordeaux et al., 2020). This CNS toxicity does not appear to be specific to the DRG. One study found cerebellar toxicity was seen after high dose IT-CM administration of AAV9 (Samaranch et al., 2014). The same study found histologic evidence of significant inflammation of the putamen after dIC injection of AAV2 with convection enhanced delivery (Samaranch et al., 2014). These studies together suggest that while injecting AAVs past the BBB may result in significantly less systemic toxicity, at high doses, this may be replaced by CNS toxicity. This suggests that additional measures beyond improving delivery past the BBB may be necessary modulate the inflammatory response to AAV gene therapy.

In addition to CNS toxicity, another major concern with IT delivery is an uneven distribution throughout the CNS. IT-L injection is the least invasive means of IT delivery generally provides good expression in the spinal cord (Bey et al., 2017; Hinderer et al., 2018a; Marshall et al., 2018) but results in little expression in the brain (Bey et al., 2017; Marshall et al., 2018). For this reason, the use of IT-L delivery has primarily been limited to the treatment of disorders involving the peripheral nerves and spinal cord (Rosenberg et al., 2018). One study used real-time MRI monitoring with co-injection with Gadoteridol contrast to estimate AAV delivery after IT-IL, IT-CM, and IT-ICV delivery (Ohno et al., 2019). They found that the IL route resulted in a broad spinal cord but little brain delivery, IT-CM delivery allowed broad distribution throughout the brain and spinal cord, and IT-ICV delivery allowed delivery to the brain only. Consistent with this, other studies also demonstrate broad CNS distribution with the IT-CM route (Bucher et al., 2014; Hinderer et al., 2014, 2018a; Bey et al., 2017; Rosenberg et al., 2018; Ohno et al., 2019).

Although IT-CM does seem capable of broad brain delivery at high enough doses, it may not do so optimally. IT-CM delivery is dependent on the natural flow of CSF through the subarachnoid space (Johanson et al., 2005). The choroid plexus of the lateral ventricles makes CSF (Ohno et al., 2019) which then flows caudally, down the third ventricle, passing through the cisterna magna, then the further to the spinal cord (Johanson et al., 2005). Though a portion of the CSF ascends rostrally, CSF’s net flow remains rostral to caudal, gently pushing intrathecally delivered drugs caudally to the spinal cord (Johanson et al., 2005). The part that ascends to the brain is, in part, drained to the facial lymphatics and into the systemic circulation (Johanson et al., 2005). This flow pattern favors the delivery of vectors to the lower brainstem, cerebellum, upper spinal cord, and caudal/lateral cortex. With IT-CM the brain’s rostral cortex and deep structures (such as the hippocampus, midbrain, and striatum) are exposed to a much lower concentration of drug (Taghian et al., 2020). Rosenberg et al. (2018) reported considerable variability in regional uptake after IT-CM injection, with the highest uptake in the posterior brain (near the injection site) and less in the cerebral cortex.

Another limitation of IT-CM delivery is that it involves inserting a needle within millimeters of the medulla (Samaranch et al., 2016; Taghian et al., 2020), posing a risk of brain stem injury resulting in permanent disability or death. Few practitioners are trained in IT-CM delivery, and optimized tools to predictably ensure the safety of patients have yet to be developed.

Interestingly, one study pioneered a new method for IT drug delivery (Taghian et al., 2020) which allows the distribution of IT-CM delivery with invasiveness of IT-L. These authors used an IA catheter, introduced via a typical lumbar puncture, and advanced this catheter to the cisterna-magna (Taghian et al., 2020). This method reduces the risk of directly accessing the cisterna magna and may allow enhanced delivery to the lower spinal cord by performing a second infusion into the lumbar region prior to removing the catheter (Taghian et al., 2020). This method was well tolerated, first in sheep, and then in two human subjects with Tay-Sachs disease (Taghian et al., 2020).

Overall, IT (and especially IT-CM) delivery of AAVs for gene therapy provides a promising alternative to dIC or IV routes. Unlike these traditional routes, IT-CM delivery provides high efficiency, non-localized brain delivery, which is not impeded by the BBB. As discussed, this route is limited by CNS toxicity at high doses, and an uneven CNS delivery of vectors throughout the brain, as well as the potential risks of injecting directly into the cisternal space. These challenges could be overcome with technological advancements. Alternatively, other routes of delivery may eventually be capable of high efficiency delivery past the BBB, and ideally with the benefits as IT-CM but without these limitations.

## Advanced Intravascular Delivery

### Intra-Arterial Delivery

Intra-arterial delivery of therapeutics to the brain has been discussed since the 1950s and has generally been in the context of treatment for neurologic malignancies such as glioblastoma multiforme (GBM) (Foley et al., 2014). Since that time, IA drug delivery has occasionally been proposed (Abe et al., 2002; Joshi et al., 2008, 2015; Riina et al., 2010), but has never taken hold. In the past decade, the utilization of interventional approaches for the treatment of neurologic conditions has increased dramatically (Cox et al., 2019), mostly in the setting of neurovascular diseases such as strokes, aneurysms, and vascular malformations. As result, there have been considerable advancements in neuro-interventional technologies (Srinivasan et al., 2020). Unlike IT-CM delivery, which remains in its infancy, most large academic hospitals have neuro-interventionalists already with the skill set necessary to deliver AAVs IA.

Though IA delivery has been proposed as a strategy for gene therapy (Abe et al., 2002; Joshi et al., 2008; Hersh et al., 2016), few studies have investigated its use in animals, and it has yet to make it to human clinical trials. The studies investigating its use in animals have clearly demonstrated the ability of this route to improve delivery to brain compared to the liver (Morabito et al., 2017; Liguore et al., 2019; Yoon et al., 2020). Interestingly, this enhancement appeared greatest with vectors which crossed the BBB with high efficiency [i.e., PHP.B (Liguore et al., 2019)] and was less dramatic with less efficient vectors [i.e., AAV9 (Morabito et al., 2017)]. This can be explained because the benefit of IA delivery is that it allows the brain the “first shot” at receiving the AAV, if the AAV is not able to efficiently cross the BBB, the majority will enter the systemic circulation. Conversely, if the AAVs are able to cross efficiently, many AAVs will enter the brain on the “first pass” thereby more dramatically reducing systemic absorption. This “first pass effect” suggests that enhanced BBB crossing (either via capsid engineering or physical BBBD) and IA delivery would synergistically enhance AAV delivery to the brain providing a very powerful method to minimize systemic absorption.

Finally, IA treatments are minimally invasive, well tolerated, with complication rates estimated to range from 0 to 0.7% (Davis et al., 2015), and are already used for many other diseases involving the brain. This route may provide a happy medium between the high systemic toxicity of IV delivery and the localized and invasive delivery of dIC. The fact that few studies have investigated this method should not remove it from consideration, as further investigation is warranted.

### Physical Methods for Transient Blood–Brain Barrier Disruption

Another approach for improving intravascular delivery is to develop method(s) to disrupt the BBB. These strategies can be used in conjunction with IV or IA delivery routes and could provide a valuable approach, which has yet to be translated to AAV gene therapy in humans. The BBB is composed of endothelial cells connected by tight junctions (Shen et al., 2017; Pandit et al., 2019). Tight junctions prevent molecules from crossing via paracellular transport (Hersh et al., 2016). Methods of physical BBBD take advantage fact that these tight junctions can be transiently disrupted by separating endothelial cells from one another, with the concept that once cells are back in proximity with each other, they will reform. This allows a window in which therapeutics, such as AAVs, are not impeded by the BBB, but that allows the BBB to reform after the therapy is delivered. The two main methods for physical BBBD which will be discussed are FUS and IA mannitol (Figure 1).

#### Focused Ultrasound

Focused ultrasound uses an array of transducers, each of which produces high frequency ultrasound waves which propagate outward, linearly (Figure 1). By positioning these in a concave, ellipsoid pattern, the beams can be focused to converge on a small target area that receives up to 1000× times more energy than any other point in the path of an individual beam (Fishman and Frenkel, 2017). The area of convergence of the FUS waves can be precisely planned using MRI (Fishman and Frenkel, 2017). A portion of the energy created by FUS waves is converted to thermal energy. Application of high-intensity FUS increases the temperature of tissue rapidly if continuously applied over a period of seconds (Fishman and Frenkel, 2017). However, if the waves are used in a pulsatile fashion (pFUS), such that the tissue has time to cool in between pulses, the temperature can never be modulated to minimize tissue damage (Fishman and Frenkel, 2017). Harmless microbubbles can be injected intravenously, and act as a substrate for the energy created by FUS, causing them to oscillate (Fan et al., 2017; Fishman and Frenkel, 2017). The oscillation of the microbubbles results in collisions with endothelial cells, disrupting the tight junctions of the BBB (Sheikov et al., 2004; Hynynen et al., 2005; Fan et al., 2017). Pulsatile FUS (pFUS) has been shown to allow for safe, well-tolerated, and transient BBBD without causing permanent tissue damage (Fan et al., 2017; Fishman and Frenkel, 2017).

Focused ultrasound mediated delivery of gene therapy to the brain has been investigated in multiple animal studies. Several studies have demonstrated its use to allow enhance the delivery genes via non-viral vectors (Nance et al., 2014; Mead et al., 2017; Yue et al., 2018). More directly relevant to this manuscript, focal pFUS has been demonstrated to greatly enhance the efficiency of IV delivery AAVs (Alonso et al., 2013; Hsu et al., 2013; Wang et al., 2015; Xhima et al., 2018; Noroozian et al., 2019; Stavarache et al., 2019). There are several key findings to highlight from these studies. First, several of these studies used AAV1, AAV2 or a hybrid of these (AAV1/2). These vectors are not used for systemic delivery without BBBD because they do not naturally cross the BBB. Interestingly, these studies all demonstrate the natural ability of these vectors to preferential transduce neurons (Alonso et al., 2013; Hsu et al., 2013; Wang et al., 2015; Stavarache et al., 2019), making the appealing starting vectors for gene therapy for NDDs. Similarly, Stavarache et al. (2019) demonstrated that dose to achieve ∼80% neuronal transduction was very low–only 1 × 10^12^ vg/kg in rats which roughly translates to 2.5 × 10^10^ vg total in mice (calculated assuming a 25 g mouse). By comparison, a dose of 1 × 10^11^ vg was required to achieve similar transduction via IA PHP.B (Liguore et al., 2019) and a 1 × 10^12^ vg was required with IV PHP.B (Hordeaux et al., 2018b) both of which naturally cross the BBB (Figure 2). It is important to note, however, that the studies with FUS only provided highly efficient delivery in the sonicated region thus a larger dose would likely be required to allow global brain transduction. Finally, it is important to identify that FUS significantly enhanced efficiency even using AAV9 (Xhima et al., 2018; Noroozian et al., 2019), this suggests that physical BBBD is more efficient at allowing BBB passage then the natural mechanism employed by AAV9.

Although there have not yet been any human trials that have attempted to enhance gene delivery to the brain, there is growing evidence of its capacity to open the BBB safely supported by pre-clinical work with a variety of molecular therapies. Patients with mild to moderate AD have undergone FUS mediated BBB opening at both frontal cortex and hippocampus to explore its potential to accelerate the clearance of intracerebral amyloid (LeWitt et al., 2019; Rezai et al., 2020). There was no significant edema or bleeding among 11 treated patients. With the recent FDA approval of aducanumab (Aduhelm) it is likely that this agent will be combined with FUS mediated BBBD in the near future (de la Torre and Gonzalez-Lima, 2021). A recent study in an AD transgenic mouse has already assessed the combination of a murine aducanumab analog and BBBD using scanning FUS (Leinenga et al., 2021). A similar approach has been utilized in patients with PD dementia (Gasca-Salas et al., 2021). This recent study of five patients supports the safety of multiple repeated pounds of BBBD of a target region at the parieto-occipito-temporal junction. Although mild improvement in cognition was observed, no significant change in either amyloid or fluorodeoxy glucose by PET scan was observed. The strategy of combining FUS mediated BBBD with an approved therapeutic is currently underway with a potential DMT for PD. There is a strong association between Gaucher’s disease caused by mutant forms of the enzyme glucocerebrosidase (GCase) with PD (Riina et al., 2010). A recombinant form of normal GCase has been an FDA approved therapy for Gaucher’s disease for many years (Pastores et al., 2004) but its large molecular size prevents crossing the BBB. A clinical trial where PD patients are infused intravenously with GCase at the same time as BBBD targeted to the basal ganglia is currently in progress (see text footnote 1 identifier NCT04370665). A study of FUS mediated BBBD in four patients with ALS (Abrahao et al., 2019) support the safety of BBBD even in patients where motor cortex, a region with symptomatic neuronal dysfunction is directly targeted. There are some potential limitations to FUS. First, some have reported sterile inflammation after it pFUS (Kovacs et al., 2017, 2018a,b; McMahon and Hynynen, 2017; Sinharay et al., 2019). While this finding’s clinical significance is unclear, it does highlight that pFUS cannot yet be considered entirely benign. Secondly, the ability of FUS to selectively disrupt the BBB in a small area only is beneficial for some applications (e.g., Neuro-oncology). This poses a challenge when attempting to use FUS in the treatment of NDDs of the CNS, which are global. One possible solution to this problem is scanning ultrasound (SUS) (Pandit et al., 2019). Similar to pFUS, with SUS, patients are pre-injected with microbubbles, but instead of converging the ultrasound waves on a single point within the brain, the focus moves throughout the brain, allowing for diffuse BBB opening (Pandit et al., 2019). Several studies have utilized SUS in the context of treating NDDs. Specifically, two studies investigated the delivery of anti-amyloid (Leinenga and Götz, 2015) and anti-tau (Nisbet et al., 2017) antibodies via SUS in animal models of AD. Both found that SUS enhanced antibody delivery throughout the brain. Further investigation of pFUS and SUS for the delivery of AAVs, either coupled with IV or IA delivery may be of significant interest.

#### Intra-Arterial Delivery of Mannitol

Intravenous delivery of mannitol can be used to pull intraparenchymal water into the vascular space thereby reducing intracranial pressure (Koenig, 2018). In contrast, IA mannitol produces a higher local concentration but much more transiently. Researchers have demonstrated that by injecting a lower total dose of mannitol directly into the carotid artery, BBBD can be achieved (Chu et al., 2018, 2020; Linville et al., 2019; Srinivasan et al., 2020). The disruption of the BBB is theorized to occur *via* two mechanisms (Hersh et al., 2016; Figure 1). First, water is pulled out of endothelial cells, causing them to shrink, and second, by causing a net efflux of water from the brain into the vasculature, which causes relative vasodilation, stretching the endothelial cells (Hersh et al., 2016). These effects place physical strain on the tight junctions that connect the endothelial cells, causing them to dissociate, allowing for paracellular translocation of large molecules across the BBB (Hersh et al., 2016). Importantly, this effect has been shown to be transient, lasting on the order of hours (Chu et al., 2018, 2020; Linville et al., 2019).

Blood–brain barrier opening with IA mannitol has been existence for nearly half a century (Chu et al., 2020). Previous work confirmed that delivery of IA mannitol opens the BBB, enhancing the delivery of chemotherapeutics such as bevacizumab to treat glioblastoma (Burkhard et al., 2012). More recently, IA mannitol has been demonstrated to increase the efficiency of AAV vector delivery across the BBB, improving the efficiency of gene therapy (Foley et al., 2014). This study used intra-carotid mannitol to deliver an AAVrh10 vector containing a TPP1 transgene (the gene-deficient in Late Infantile Neuronal Lipofuscinosis). They found that animals pre-injected with intra-carotid mannitol demonstrated significantly higher TPP1 expression at the same dose than animals who received a saline. Interestingly, the vector used was AAVrh10, which *does* cross the BBB naturally, suggesting that IA mannitol allows more efficient BBB crossing than naturally occurring AAVs. A comparison of the efficiency of IA mannitol and engineered vector efficiency (such as PHP.B) has not yet been done. Unfortunately, a major limitation of this study was that 1/3 of mice receiving the high dose of IA mannitol developed “morbidity” (Foley et al., 2014), related to the observation that high concentrations of IA mannitol may result in osmotic lysis of cells (Chu et al., 2018; Linville et al., 2019). As a result, based upon these results, the method of IA mannitol delivery in this study may not be viable for human translation.

To improve the safety of IA mannitol one study used real-time MRI imaging after carotid injection of superparamagnetic iron oxide (SPIO) to determine an optimal flow rate, which would allow for widespread delivery of mannitol at the lowest possible dose (Chu et al., 2018). They found that the infusion rate (and dose) used by Foley et al. (2014) was five times higher than the optimal dose (Chu et al., 2018). The optimal flow rate varied from animal to animal, likely related to normal variation in vascular anatomy. Individualized rates of flow for each animal prior to mannitol delivery allowed for more consistent and transient BBBD without signs of cell death or inflammation (Chu et al., 2018).

One limitation observed in this study was that, at safe infusion rates, there was excellent distribution of mannitol to the deeper structures of the brain such as the hippocampus, but not to the cortex (Chu et al., 2018, 2020). In a second study, this same group, found that clamping the contralateral common carotid artery prior mannitol administration significantly improved cortical perfusion (Chu et al., 2020). In humans, this could theoretically be accomplished via brief balloon occlusion rather than a permanent clamp.

A final caveat to the above studies is that they were conducted in mice. The decision to use mice allows the methodology and parameters for mannitol delivery developed by these authors to be applied in many disease models (including NDDs), which rely on the existence of transgenic and inexpensive animal models (Chu et al., 2020). It has been shown in mice and larger animals such as rabbits that MRI image guidance is essential to predict and precisely open BBB (Janowski et al., 2016). It has been demonstrated in one case report that MRI guidance of BBB opening is also feasible in the patient (Zawadzki et al., 2019).

Intra-arterial delivery with IA mannitol offers several significant advantages that may make it an ideal method for the global delivery of AAVs past the BBB. First, it allows for *global* disruption of the BBB, which, as discussed previously, maybe a critical factor in achieving successful gene therapy for NDDs of the brain. Second, if an IA route is used, IA mannitol (delivered pre-determined, safe flow rate) would not contribute significantly to invasiveness. Last, the synergistic enhancement of the IA delivery with BBBD should allow vector delivery to the brain on the “the first pass” allowing many AAVs delivery to the brain before encountering neutralizing antibodies or the liver.

## AAV Capsid Engineering

The properties of an AAV, termed the *capsid profile* (Wec et al., 2021), includes tissue tropism, cellular tropism, efficiency, immunogenicity, and ability to cross the BBB in the case of certain serotypes (Büning and Srivastava, 2019). The capsid profile is determined by the amino acid sequence of its *cap* gene which encodes for the viral capsid (Hocquemiller et al., 2016). Manipulating the coding sequence of this gene allows us to change the biologic properties of that capsid. As BBB crossing is a significant barrier of delivery to the brain, most engineered AAVs discovered with tropism for the brain have achieved this by improving the efficiency by which the AAV penetrates the BBB (Deverman et al., 2016; Jackson et al., 2016; Hordeaux et al., 2018b, 2019). Enhancing BBB crossing alters AAV *tissue* tropism allowing AAVs improved delivery throughout the brain. Similarly, non-target tissues, such as the liver, can be de-targeted (Pulicherla et al., 2011; Choudhury et al., 2016).

Capsid engineering can also be utilized to alter the cellular tropism AAV variants. Engineered AAV variants have been developed with enhanced neuronal tropism or enhanced tropism to brain endothelial cells (Kumar et al., 2020). The potential for capsid engineering extends beyond cellular tropism as well and other strategies for mitigating the immune response including removal of neutralizing antibody binding sites and removal of highly immunogenic antigens has been proposed (Wec et al., 2021). Although we have an idea of what AAV properties are desirable, determining capsid alterations at a sequence level, which would provide novel properties, in humans, poses a challenge. Ideally, one could employ rationale design and make a change that is known to have a targeted effect. Unfortunately this method is challenging using current knowledge and technologies (Bell et al., 2012). In order to overcome this problem, researchers have developed methods based on the same strategy nature has used, namely “evolution” (Pulicherla et al., 2011; Deverman et al., 2016; Chan et al., 2017; Büning and Srivastava, 2019; Kumar et al., 2020; Nonnenmacher et al., 2021).

### Directed Evolution of AAV Capsids With *in vivo* Selection

Directed evolution of AAV capsids has now become a key strategy underlying efforts to develop AAV variants with optimized properties (Büning and Srivastava, 2019). At its core, directed evolution has two major steps; random mutagenesis then targeted selection. Over the past 10 years, researchers have pioneered methods for evolving AAV capsids to suit specific purposes in mice. Engineering capsids for humans will likely require further maturation of this technology, but the many principles underlying the methodology remain the same. For that reason, a selection of key studies which advanced the field of capsid engineering will be reviewed. After this we will discuss possible strategies for building upon these methods for human translation.

Pulicherla et al. (2011) were the first to use random mutagenesis and targeted selection to identify AAV capsid variants with an improved property for CNS gene therapy, in this case liver de-targeting. These authors used error-prone PCR to generate a library of AAV9 variants containing a luciferase transgene. Variants were then injected systemically, and bioluminescence was used to determine their geographic biodistribution in live animals (Pulicherla et al., 2011). This allowed the authors to identify two variants with decreased tropism to the liver, which they called AAV9.45 and AAV9.47 (Pulicherla et al., 2011). Later, a different study built on this discovery by using AAV9.47 as a parent for another modification designed to enhance delivery to the brain (Choudhury et al., 2016). These authors found that the addition of 19 alanine residues to the N-terminus of VP2 improved neuronal tropism. The resulting vector, AAV-AS was the first to be created with multiple desirable properties for CNS gene therapy, liver de-targeting, and enhanced neuronal tropism (Choudhury et al., 2016).

Deverman et al. (2016) published a landmark paper introducing a technique which they called Cre recombination-based AAV targeted evolution (CREATE). In this method, a large number of randomly generated peptide inserts are placed within the variable region of a VP to create a library of modified AAV capsids. Next, these are injected, intravenously into Cre-transgenic mice. A Cre-lox system is used to limit expression of a reporter gene to cells which successfully transduced target cells. Transgenes, which include *cap* gene of each variant are recovered from target tissue and sequenced to identify variants with the highest efficiency for target cells (Deverman et al., 2016). Using this strategy, they to developed the novel capsid, AAV PHP.B, which demonstrated a 50–100 fold increase in the CNS uptake, after IV injection, compared to AA9 (its parent) (Deverman et al., 2016). In later studies, reverse engineering of PHP.B allowed researchers to determine that it’s enhanced CNS tropism was due to interaction with the receptor LY6A (Hordeaux et al., 2019). LY6A is a GPI-anchored protein, present on lipid rafts of endothelial cells (Huang et al., 2019), and is highly expressed in brain microvasculature. Binding of PHP.B to LY6A allowed it to have the novel property of highly efficient BBB crossing. Unfortunately, further studies determined that this property was specific to the species in which it was created, C57 mice, and its ability to transduce the CNS in other species, including rhesus macaques (Hordeaux et al., 2018b; Liguore et al., 2019), marmosets (Matsuzaki et al., 2018), rats (Dayton et al., 2018), and even a different strain of mice (BALB/cJ) (Hordeaux et al., 2018b), was not different than AAV9.

Later, the same group used PHP.B as a parent, then conducted a second round of CREATE, again selecting variants from the CNS. Through this second route of CREATE, they were able to identify a variant with roughly 2.5 greater enhancement of CNS transduction compared to PHP.B, which they named PHP.eB (Chan et al., 2017). This study demonstrated the potential of using multiple rounds of directed evolution to further enhance the same property.

Another group developed a method similar to CREATE, except in reverse. In this method, which they called *iTransduce*, the gene delivered contains the Cre sequence and AAVs were injected into transgenic animals expressing a loxed reporter (Hanlon et al., 2019). They then used flow cytometry to sort the cells expressing the reporter, recovered the capsid DNA from them, and sequenced it. Using this method, these authors identified a unique capsid with high tropism to the CNS similar to PHP.B, which they called AAV-F (Hanlon et al., 2019). Interestingly, unlike PHP.B, they found that AAV-F was able to transduce BALB/cJ mice. They indicated that a future direction of this study was to test this vector in large animal models. However, to our knowledge, this has yet to be published.

Finally, the same group that introduced CREATE built upon their method, publishing an improved technique which they called multiplexed CREATE (or M-CREATE) (Kumar et al., 2020). M-CREATE improved on the initial method by using several techniques to reduce experimental bias generated by the first round of selection, followed by a second round (full explanation of their methods is available in their paper). Implementation of these methods allowed the author to identify *many* more AAV variants with broadly enhanced CNS tropism, including several vectors (AAV-PHP.C1, C2, and C3) with retained properties in BALB/cJ mice. Interestingly, by using cell-specific promotors to drive cre expression in transgenic animals the authors also identify a novel vector with decreased glial cell tropism but retained, highly efficient, neuronal transduction (PHP.N).

### Strategies to Improve Capsid Engineering for Brain Delivery in the Future

The studies described in the previous section, and others related studies, have been important for the advancement of the field of AAV capsid engineering. Despite this, there are several major limitations which have yet to be overcome. Most notably, the lack of across species translation. While methods like CREATE are directly applicable to evolving variants with properties in mice for research (Haery et al., 2019), their ability to discover variants with clinical significance is limited by the model in which they are screened (Hordeaux et al., 2018b). All of the methods for directed evolution discussed thus far screen variants *in vivo*.

There are several significant limitations of *in vivo* screening of variants including: (1) It is not possible to screen variants *in vivo*, in humans, therefore this method is not sufficient to identify variants with optimized properties in humans, and (2) Compared to *in vitro* or *in silico* methods for screening, *in vivo* methods are slow, expensive and labor intensive (Wec et al., 2021). Prior reports have confirmed that multiple rounds of evolution yields variants with greater efficiency at achieving the same property (Dayton et al., 2018). As such, the utilization of screening methods which can realistically be repeated many more times would be preferable. Finally, (3) *In vivo* screening selects variants based on the sum of their abilities to reach a target. Every variant, therefore, must possess every trait necessary to achieve efficient gene delivery in that model organism. Screening for all properties, in *parallel*, selects variants with the highest *overall* fitness however its ability identify optimal variants with *specific* properties is limited. Because variants possessing novel properties are rare (Wec et al., 2021), large, diverse libraries are likely required to find the best variants (Bryant et al., 2021). When using multiple rounds of evolution, screening *in vivo* greatly limits the diversity of libraries used in subsequent generations. This is because all variants identified must possess the small subset of traits which confer the greatest overall enhancement of fitness (e.g., BBB crossing) even if this property is not felt to be the most important.

**FIGURE 4:**
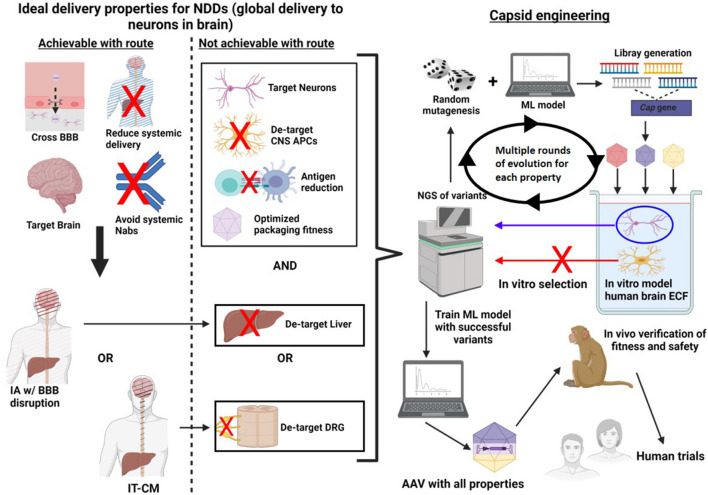
A method of directed evolution to optimize AAV delivery to the brain, in humans. First, properties of an idea delivery system are identified. Next, a delivery route is chosen which optimizes as many of these properties ill and of itself. Capsid engineering can be used to acquire as many of the remaining properties as possible. To do this, random mutagenesis is used and a ML model trained to select only variants with high packaging fitness can be used to diversify the library. Next these variants are introduced into an *in vitro* model which selects for a desired property, for example a model containing all of the main cell types which the virus could transduce. The DNA is then harvested from the target condition and sequenced using next generation sequencing. In this example variants which transduced neurons are identified for positive selection, while variants which transduced APCs are identified for negative selection. The ML model is then trained with the lists of successful variants. This process is repeated multiple times for a single property until novel high yield variants are no longer discovered in successive rounds of evolution. Once one property is complete, this process can be repeated for new properties using a different *in vitro* model for selection. Finally, the ML algorithm which has been trained with all of the data can identified variants which have the greatest fitness for all selected properties. These variants are then introduced into NHPs *in vivo* to confirm that they are function in a live environment and that they are safe. Finally, the variants are ready for human trials.

Instead of starting with *in vivo* screening, other methods, including *in vitro* (in an artificial environment) (Wec et al., 2021) and *in silico* (in a computer) (Bryant et al., 2021; Marques et al., 2021; Wec et al., 2021) screening can be used (Figure 4). *In vitro* screening is advantageous because it allows the use of human cells for selection, and is it allows targeting of specific properties by changing the selection environment. Targeting specific properties, *in serial*, would be beneficial because it allows the generation of libraries focused on a target property thereby increasing the statistical likeliness of its discovery. Additionally, there are likely a finite number properties which can be optimized in one capsid before the sequence alterations interfere with each other or with packaging fitness (Wec et al., 2021). For this reason, selection *in serial* allows the addition of new properties while ensuring the original properties are retained.

*In vitro* screening requires the generation of a model environment which re-capitulates the scenario underlying the desired property (Figure 4). For example, if the goal is to identify capsid variants with enhanced neuronal entry, one could model the extracellular environment of the human brain, including target cells (neurons) and non-target cells (astrocytes, microglia etc.). AAV variants could then be introduced into the model, and variants expressed within neurons can be selected and sequenced. The variants obtained could then be subjected to further randomization and the process could be repeated many times, ultimately the yield should be variants with high human neuronal tropism.

There are several key limitations to *in vitro* screening. First, though less of a problem than with *in vivo*, each round of *in vitro* selection still requires a significant amount of time and resources, limiting the number of possible rounds of evolution. Second, using random mutagenesis alone to generate new libraries creates many “dead-end” variants, which either lose the ability to be produced, or lose the property of interest. Fortunately, *in silico* methods, should overcome these concerns. *In silico* screening methods utilize machine learning (ML) algorithms to make mathematically driven predictions about physical processes (Wec et al., 2021). Similar to *in vivo* screening methods, *in silico* screening does not require a mechanistic understanding of the processes involved to make accurate predictions (Brown et al., 2017; Bryant et al., 2021; Marques et al., 2021; Wec et al., 2021). In order to make these predictions, data must first be supplied to the ML model in a process known as training. Data for training ML algorithms can be obtained, by conducting *in vitro* selection of randomly generated libraries. The sequences successful and unsuccessful variants can then be supplied to the ML algorithm allowing it to then predict successful variants in the future (Bryant et al., 2021; Wec et al., 2021).

Machine learning models can be used at multiple steps of capsid engineering. Two studies have recently been published demonstrating methods for utilizing ML, to generate libraries with high diversity (large number of mutations) and retained packaging viability (Bryant et al., 2021; Marques et al., 2021). These developments are valuable because variants with a novel property may differ significantly from the parent capsid, thus to optimize discovery large libraries with extensive mutations may be necessary (Bryant et al., 2021). Another use for ML, in capsid engineering, is to screen newly identified variants for a given property to ensure packing viability, and all prior properties are retained. Once multiple rounds of evolution have identified a small number of variants with as many optimized properties as possible, these final variants could be subjected to *in vivo*. Ideally, this step would be done in NHPs to best recapitulate human physiology. That said, the comparative efficiency of these variants in NHPs may be not reflective of their efficiency in humans as the variants were screened in human cells. For this reason, this final step would be to confirm that variants are able to reach the brain safely (regardless of final titer).

It is recognized that the above strategy for capsid engineering is theoretical and unvalidated. That said, it is based on the same conceptual framework as other, successful methods of directed evolution, involving the same two key steps of random mutagenesis and targeted selection. The only differences are the model in which variants are screened and the use of ML algorithms for improving library development which have been validated in prior studies (Bryant et al., 2021; Marques et al., 2021). It is hoped that this method could be a novel method which would allow capsid engineering directly applicable to human translation, something which has not yet been achieved.

## Improving Specificity of Transgene Expression

The final step in AAV delivery is the actual expression of transgenes. Modifying the location or context in which the transgene expressed allows a powerful tool which can enhanced the selective of a gene therapy. One method for altering transgene expression is the choice of promoter. Promoters dictate which cells, under what conditions, and with what strength, a transgene will be expressed (Jackson et al., 2016). Currently, most gene therapies use a ubiquitous promoter that is constitutively active in all cells. Commonly used ubiquitous promoters are cytomegalovirus (CMV), chicken β-actin (CBA), human elongation factor 1 alpha (EF1α), and variants of these (Bedbrook et al., 2018). Zolgensma^®^, for example, uses a CBA promoter (Hoy, 2019). The benefit of a ubiquitous promoter is that it allows for the highest level of overall transcription (Ingusci et al., 2019). This is especially useful when the vector itself is inefficient as a strong promoter allows for higher gene expression even if relatively few cells are transduced (Jackson et al., 2016).

There are several problems with ubiquitous promoters. The first is that long-term unregulated overexpression of a transgene can result in unintended alterations in cellular signaling (Ingusci et al., 2019). Second, transgenes can have undesirable effects when expressed in a tissue outside of that which it is intended (Ingusci et al., 2019). Finally, the adaptive immune response to gene therapy products can be induced by the transgene product itself in a dose-dependent manner (Samaranch et al., 2014; Perez et al., 2020). Ubiquitous expression in off-target cell types would increase the risk of immune-mediated toxicity.

There are many cell-specific promoters with relevance to gene therapy for NDDs. Promoters can limit expression to a specific brain cell type (Jackson et al., 2016; Bedbrook et al., 2018), brain structure (Chan et al., 2017) or cells producing a specific neurotransmitter (Bedbrook et al., 2018; Ingusci et al., 2019). As the primary site of pathophysiology in NDDs are neurons, neuronal promotors would be optimal for gene therapy in these conditions. The human synapsin (hSyn1) promoter has been demonstrated as an effective neuronal promoter when used with AAVs (Jackson et al., 2016; Bedbrook et al., 2018). One study compared CNS expression of 4 different promotors–hSyn1, human CMV, mouse PGK (mPGK) and short variant of CMV early enhancer/chicken beta actin (sCAG)–each driving expression of GFP delivered via intraparenchymal injection of AAV1 in mice. They found that significantly higher expression with hSyn1 and mPGK compared to sCAG and CMV shortly after the injection (Jackson et al., 2016). Conversely, Jackson et al. (2016) found that expression with synapsin waned after 4–5 months while expression with CMV/Chicken beta actin (CBA) did not. Further investigation into persistence of hSyn expression, ideally in humans, will be important to ascertain its viability.

A novel use of promoters, which, to our knowledge has not yet been described, can be called “environment-specific” promoters. These can be defined as promoters that drive transgene expression only under specific cellular conditions. One strategy would be to use a promoter for proteins specifically activated in the context of proteostatic stress (e.g., in cells nearing neurodegeneration). In this strategy, the transgene would lay dominant in target cells until these cells are under proteostatic stress. As such transgene toxicity could be avoided unless a cell is already going to undergo neurodegeneration. One example of how this could be accomplished uses a response called the unfolded protein response (UPR) (Valenzuela et al., 2018). In this response, proteostatic stress is detected by stress transducers in the ER lumen. These transducer molecules activate receptors, which activate transcription factors such as ATF6f, XBP1s, and ATF4 (Valenzuela et al., 2018). The transcription factors then activate genes whose products can reduce proteostatic stress by a variety of mechanisms (Valenzuela et al., 2018). Because this signaling cascade is capable of identifying when a cell is under proteostatic stress, and is able to use that information to active the expression of specific genes, the promoter which drives these genes could be ideal for gene therapy for NDDs. This would be especially true if the transgene is designed to knock down a protein that, under physiologic conditions, serves a vital cellular function. A similar concept of tumor-specific promoters, has been proposed in the oncology literature (Lu et al., 2005).

Another interesting strategy is synthetic promoters. Synthetic promoters are artificially designed promoters comprised of multiple transcription factor regulatory elements (TFREs) (Brown et al., 2017). These elements may be designed to bind multiple endogenous transcription factors, increasing the sensitivity of the system (Brown et al., 2017). Artificially created promoters can be engineered for enhanced long-term gene expression by removing CpG motifs targeted for methylation-mediated silencing (Brown et al., 2017). Further, targeted mutagenesis and directed evolution can also be used for to improve the efficiency of artificial promoters. One group generated a library of DNA sequences that fused to the 5′ of an artificial promoter and identified a modification that led to a 5.8 fold increase in promoter strength (Jin et al., 2019). Similar to capsid engineering, directed evolution with *in vitro* and *in silico* selection methods could allow for the discovery of promoters with optimized properties far beyond those which are naturally occurring.

Finally, manipulations to enhance the specificity of transgene expression is not limited to promoters. Several groups have demonstrated the effectiveness of incorporating cell-specific miRNA binding sites into the transgene. Sequences which bind miRNA produced in off target cells can be chosen. This method allows for specific knock down in cells with undesirable consequences to transgene expression, for example APCs (Piguet et al., 2021).

## Transgene Persistence After Delivery

Re-administration of viral vector gene therapies poses a major risk. After the first treatment with a viral vector, patients will likely develop memory B and T cells to the virus (Rabinowitz et al., 2019; Verdera et al., 2020). If this occurs, a greater adaptive immune in response to any subsequent doses would be expected, greatly increasing the risk of immune-mediated cytotoxicity and/or neutralization by antibodies generated after the initial treatment. While the risk of immune reaction to re-administration is lower with gene therapies which do not require a viral vector, such as antisense-oligonucleotides, the requirement of repeated IT administration (Drahansky et al., 2016) has its own set of risks and challenges.

The ideal gene therapy would be one that requires only a single administration to achieve life-long therapeutic benefit. This should be possible with DNA-based gene therapies, which reside in the nucleus and can be continuously expressed for the life-time of that cell. Though this is theoretically possible, transgene persistence has been a major issue faced by DNA-based gene therapies. Two tong terms follow up studies of patients treated with AAV gene therapy have recently in the hemophilia literature. In one, patients were followed up 3 years after treatment of Hemophilia A with AAV5 delivered human factor VIII gene (Symington et al., 2021). They found that factor VIII activity waned after the first year in most patients. Interestingly, though gene expression had decreased considerably, the clinical benefit of the therapy was sustained through the 3 years of investigation. In the second study, in patients with hemophilia B followed 8 patients after treatment with a “codon-optimized” human factor IX gene delivered by AAV8 (Konkle et al., 2021). Only 1 of 8 patients had sustained factor IX activity at 4 years post-treatment. The authors explain that their codon-optimization program inadvertently added a large quantity of CpG sequences. They hypothesized that their transgene loss was due stimulation of an innate immune response related to these added sequences (Konkle et al., 2021).

In the case of the CNS, Onasemnogene Abeparvovec (Zolgensma^®^) is the only DNA-based gene therapy delivered to humans long enough ago to evaluate transgene persistence. Of the patients treated with Zolgensma^®^, all had, at minimum, maintained the same motor function (many had developed new milestones) and remained off mechanical ventilation up to 6.2 years post-treatment (Mendell et al., 2021). This is especially noteworthy in SMA type 1 where the natural history of the disease results in 92% of patients requiring mechanical ventilation by 20 months of age (Mendell et al., 2017). Although Mendell et al. do not provide direct evidence supporting persistence of their transgene (i.e., measurement of SMN activity) their clinical results suggest persistence of transgene activity in their patients.

Although the results from Mendell et al. are encouraging, waning transgene expression remains a major concern which should be optimized in any way possible. Consideration of optimizing transgene persistence should start with identification of potential mechanisms by which transgene loss can occur. Proposed mechanisms include (1) loss of transgene expression due to immune response (Mulia et al., 2020; Konkle et al., 2021; Symington et al., 2021), (2) episomal loss due to cellular turnover without transgene replication (Lufino et al., 2008; Mulia et al., 2020), (3) epigenetic modifications resulting in gene silencing (Mulia et al., 2020). Loss of transgene expression due to the immune system has been proposed to occur either by adaptive or innate immune responses. CpG sequence activation of TLR-9 has been implicated in the innate response while, transgene loss due to an adaptive response is thought to be due to T-cell mediated cytotoxicity (Konkle et al., 2021). In either case, “transgene loss” would be the result of destruction of the cells harboring the transgene (rather than isolated loss of a transgene in an otherwise intact cell). In the liver, cell loss due to immune destruction may be clinically silent as hepatocytes are capable of replicating to replace lost cells. However, as neurons are largely incapable of replication, immune-mediated transgene loss therefore causes permanent neuronal loss synonymous with immune-mediated neurotoxicity (Wang et al., 2019). This process has been demonstrated to irreversible and mediated by cytotoxic T-Cells, IFN-γ, TNF-α, and perforin (Wang et al., 2019). Strategies to counter immune-mediated toxicity (and therefore transgene loss) are a major focus of earlier sections in this manuscript and will not repeated here.

With AAV vector delivery, the majority of delivered transgenes will exist as an episome, rather than integrating into the host genome. This can be beneficial, as it reduces the risk of insertional mutagenesis, however, unless extra measures are taken, the transgene will not replicate with rest of the genome, overtime, transgene expression will naturally be lost (Mulia et al., 2020). Though this is a major challenge in most other organs, the highly limited neuronal replication is beneficial, in this case, and this problem may not need to be addressed when considering gene therapy for diseases predominantly involving neurons.

Epigenetic silencing poses a threat to delivered transgenes (Lufino et al., 2008; Mulia et al., 2020). There are several means to combat this. First, it has been demonstrated that viral promoters, such as the CMV promoter, are subject to greater rates of silencing (Mulia et al., 2020) thus mammalian promoter (ideally human) should be preferentially considered. Second, specific sequences (e.g., CpG sequences) are known to be sites of gene silencing (Mulia et al., 2020; Konkle et al., 2021), minimization of these, and other sequences known to be targeted for methylation should be considered to improve transgene persistence. Finally, extrachromosomal DNA (i.e., episomes) are less subject to silencing compared integrated DNA (Lufino et al., 2008). For this reason, vectors such as AAVs which rarely integrate into the host genome would be preferred to avoid epigenetic silencing.

Overall, the length of persistence of episomal transgenes after AAV gene therapy is not known, especially in the case of treating neurologic diseases. While there is evidence of persistent clinical benefit for at least 6 years, it is unclear how long this might last. As there was no quantitative measurement of SMN it is unclear if the clinical Zolgensma^®^ has reached its full potential, or if it is being blunted by waning gene expression. Future studies investigating transgene expression over time after CNS gene therapies would be valuable to address these concerns, however it is noteworthy that this would likely require repeated spinal taps to obtain CSF.

## Conclusion

Gene therapy provides a logical means for treating many neurologic diseases. NDDs are especially amenable to gene therapy as the pathogenesis of most is mediated either by dysfunction of a single gene or by a single, or small number of proteins (Kovacs, 2019). Gene therapy delivery to the brain can be accomplished using AAV vectors. However, these vectors, and the transgenes they carry, pose significant risks to patients, especially when delivered at high doses. Optimizing the efficiency with which AAVs are delivered is critical to the success of these therapies. This manuscript discusses a subset of AAV delivery technologies thought to contribute to our vision of the ideal gene therapy for the treatment of NDDs of the brain. The vision is to have a single dose gene therapy delivered globally to the brain, with minimal systemic delivery. This therapy would preferentially target neurons, and avoid immune cells such as APCs. Finally, and importantly, the delivery of the therapy does not require opening of the skull. To accomplish this vision, we consider each step of AAV delivery individually, and highlight methods which can improve delivery at that step. We envision a multifaceted approach for optimizing AAV delivery to the brain, which, if successful, would allow for the translation of gene therapies for the treatment of many diseases of the brain.

## Data Availability Statement

The original contributions presented in the study are included in the article/supplementary material, further inquiries can be directed to the corresponding author/s.

## Author Contributions

JF did most writing and all figures. PF provided the regular guidance, education, suggestions and edited the multiple revisions. Both authors contributed to the article and approved the submitted version.

## Conflict of Interest

The authors declare that the research was conducted in the absence of any commercial or financial relationships that could be construed as a potential conflict of interest.

## Publisher’s Note

All claims expressed in this article are solely those of the authors and do not necessarily represent those of their affiliated organizations, or those of the publisher, the editors and the reviewers. Any product that may be evaluated in this article, or claim that may be made by its manufacturer, is not guaranteed or endorsed by the publisher.

## Footnotes

1
clinicaltrials.gov
